# A systematic review to establish the frequency of cyclooxygenase-2 expression in normal breast epithelium, ductal carcinoma *in situ*, microinvasive carcinoma of the breast and invasive breast cancer

**DOI:** 10.1038/bjc.2011.204

**Published:** 2011-06-07

**Authors:** J A Glover, C M Hughes, M M Cantwell, L J Murray

**Affiliations:** 1 Centre for Health Improvement, Queen's University, School of Medicine, Dentistry and Biomedical Sciences, Institute of Clinical Science Building, Block B, Royal Victoria Hospital, Grosvenor Road, Belfast BT12 6BA, UK; 2 Centre for Health Improvement, Queen's University Belfast, School of Pharmacy, Medical Biology Centre, 97 Lisburn Road, Belfast BT9 7BL, UK; 3 Centre for Public Health, Queen's University, School of Medicine, Dentistry and Biomedical Sciences, Institute of Clinical Science Building, Block B, Royal Victoria Hospital, Grosvenor Road, Belfast BT12 6BA, UK

**Keywords:** breast cancer, COX-2, Cyclooxygenase-2, DCIS, MICB, review

## Abstract

**Background:**

Epidemiological studies have suggested a protective effect of cyclooxygenase (COX)-inhibiting non-steroidal anti-inflammatory drugs in breast cancer risk and disease progression. We performed a systematic review to evaluate the frequency of COX-2 expression in normal breast epithelium, ductal carcinoma *in situ* of breast (DCIS), DCIS-adjoining invasive breast cancer, microinvasive carcinoma of the breast (MICB) and invasive breast cancer.

**Methods:**

Literature searches were carried out on MEDLINE, EMBASE and Web of Science from their commencement until September 2010. Primary studies examining COX-2 expression by immunohistochemistry methodology were included. Meta-analyses were carried out using random effects models for individual study estimates of COX-2 expression and pooled to give an overall estimate.

**Results:**

The pooled prevalences (95% confidence intervals) of COX-2 expressions were 53% (44–61) in DCIS studies and 42% (36–49) in the invasive breast cancer studies. There were too few studies involving normal breast epithelium, DCIS-adjoining invasive breast cancer and MICB to conduct meta-analyses.

**Conclusion:**

The findings from our meta-analyses have shown similar COX-2 expression in DCIS and invasive breast cancer. This may suggest the involvement of COX-2 in early carcinogenesis. Further studies of COX-2 expression in DCIS are required to investigate the use of COX-2 as a potential drug target for prevention of disease progression in DCIS.

The cyclooxygenase (COX) enzyme system consists of two forms: COX-1 and COX-2. COX-1 is constitutively expressed, whereas COX-2 expression is induced by cytokines, growth factors and oncogenes (Howe, 2007). There is considerable evidence to suggest a role for COX-2 in carcinogenesis. The prostaglandins produced by COX-2 are involved in key processes in cancer development including proliferation, mutagenesis, resistance to apoptosis, angiogenesis, immune suppression and invasion (Singh and Lucci, 2002; Harris, 2009). It has been proposed that COX-2 expression in ductal carcinoma *in situ* (DCIS) has a crucial role in the development of breast cancer, with the ability of DCIS to progress to invasive breast cancer being dependent on the COX-2 enzyme's role in the destruction of the basal membrane and in the formation of new blood vessels allowing tumour growth (Imada *et al*, 2006). COX-2 expression in invasive breast cancer tissue has been widely documented and expression has been associated with poorer prognostic parameters and disease progression (Singh-Ranger *et al*, 2008). The use of COX inhibitors has been associated with reduced breast cancer risk (Takkouche *et al*, 2008). In addition, several studies have suggested a reduced risk of recurrence and death from breast cancer in women who have used non-steroidal anti-inflammatory drugs (NSAIDs) after diagnosis (Blair *et al*, 2007; Kwan *et al*, 2007; Holmes *et al*, 2010). However, these studies have not examined tumour COX-2 expression in relation to disease progression.

To further explore the role that COX-2 expression may have in the development and progression of breast cancer, we undertook a systematic review to determine the frequency of COX-2 expression in the continuum from normal breast epithelium to invasive carcinoma of the breast.

## Methodology

### Literature search

Systematic searches were conducted using three electronic databases (MEDLINE, EMBASE and Web of Science) from their commencement to September 2010. Identified keywords and medical subject heading (MeSH) terms for COX-2 were combined using the ‘OR’ operator. This was repeated for normal breast epithelium, DCIS, microinvasive carcinoma of the breast (MICB) and invasive breast cancer. Results for COX-2 and either normal breast epithelium, DCIS, MICB and invasive breast cancer were then combined using the ‘AND’ operator (Supplementary Resource 1 online). The search strategy was limited to human studies and where publications were available in English.

Duplicate articles were removed and titles, and abstracts if necessary, were screened to exclude irrelevant articles. Where an abstract was not available or the article's significance was unclear, the full article was examined. Articles retrieved for DCIS (DCIS-adjoining invasive breast cancer) and MICB were independently screened for relevance by three reviewers (CH, LM and JG) and discrepancies were reviewed by a fourth person (MC). Articles retrieved for normal breast epithelium and invasive breast cancer were independently screened by two reviewers (JG and either MC, CH, LM or colleagues) and discrepancies were discussed as a group (MC, CH, LM and JG). References of all included articles were screened to identify other potentially relevant publications (JG).

Articles were assessed according to predefined inclusion and exclusion criteria. For inclusion, studies were required to be primary investigative research, have used an immunohistochemical (IHC) method for the assessment of COX-2 expression and have reported the proportion (%) of breast tissue samples exhibiting COX-2 expression or data from which this proportion could be calculated. As monoclonal antibodies are more specific, have a higher homogeneity and therefore provide more reproducible results than polyclonal antibodies, only studies using monoclonal anti-COX-2 antibodies were included (Abcam plc, 1998–2010). Assessment of staining in IHC studies is subjective therefore only studies where COX-2 expression was evaluated using at least two independent assessors were included. Articles that included samples from men or from women with a previous history of DCIS or invasive breast cancer were excluded. Studies of either normal breast epithelium, DCIS or DCIS adjacent to invasive cancer and MICB that contained fewer than 10 samples were excluded. As a greater number of studies were published on invasive breast cancer, studies that contained fewer than 100 samples were excluded. For studies in which the sample source was reported on repeatedly, only the largest set of results was used. Of note, a variety of definitions have been applied to MICB in relation to the extent of stromal invasion (Bianchi and Vezzosi, 2008). We included any definition of MICB in this review.

### Data extraction

Data extraction was performed by the principal reviewer (JG). Data extraction forms were designed to record information on the year of publication, study location, source of data, number of samples, time period over which samples were obtained and the method of evaluation of COX-2 expression.

### COX-2 evaluation

The evaluation method for COX-2 expression differed between studies. Several studies provided an immunoreactive score (IRS), which was calculated by multiplying a staining intensity score with a score for quantity of staining. Other studies only assessed the quantity of COX-2 staining. Only lesions reported to express COX-2 (by either of these methods) at a moderate or strong level were defined as COX-2 positive within this meta-analysis. A further description of COX-2 evaluation in the studies is provided in Supplementary Resource 2 online.

### Statistical analysis

We used random effects models to account for both within-study and between-study variance. Individual study estimates (ES) for COX-2 expression from each study were pooled to give an overall estimate. All estimates were calculated with 95% confidence intervals (CIs). The degree of heterogeneity among studies was investigated for each tissue group using *χ*^2^-tests (Q statistic) and the I-squared measure (I^2^ statistic). Forest plots were used to depict the results of pooled analyses. All statistical analyses were carried out using the ‘meta’ package in STATA (SE version 11.0; StataCorp 2005, College Station, TX, USA).

## Results

### Normal breast epithelium

The search identified 833 studies (Medline *n*=47, Embase *n*=105 and Web of Science *n*=681). A total of 800 articles were screened after removal of duplicates. A total of 24 potentially relevant papers were fully screened. Additionally, one paper was included from the DCIS search results. In all, 23 studies were excluded as the samples were taken adjacent to breast cancer or from patients who previously had breast cancer (*n*=11), the proportion (%) of COX-2 expression was not reported or could not be calculated (*n*=9), the study did not use monoclonal anti-COX-2 antibodies (*n*=2) or did not report independent evaluation of COX-2 staining by two assessors (*n*=1). Two studies, one Korean and the other from the United States of America, met the inclusion criteria and meta-analysis of the data was not possible. Both studies were hospital based and samples were extracted from tissue archives. Similar COX-2 expression evaluation methods were reported. The prevalences of COX-2 expression in these two studies were 6.7 and 100% (Table 1). The time period for which breast tissue was obtained was not specified in either study, and only one study reported that the samples were from reduction mammoplasty (Zhao *et al*, 2008).

### DCIS

**Figure 1:**
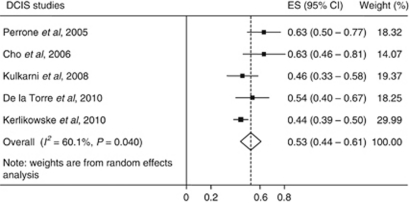
Forest plots of COX-2 expression in DCIS.

The search identified 303 studies (Medline *n*=40, Embase *n*=61 and Web of Science *n*=202). A total of 275 articles were screened after removal of duplicates. A total of 36 potentially relevant studies were fully screened. In all, 31 studies were excluded as the proportion (%) of COX-2 expression in DCIS was not reported or could not be calculated (*n*=13), the sample size was below 10 (*n*=6), the tissue source was used in a larger study (*n*=9), the study did not state use of monoclonal anti-COX-2 antibodies (*n*=2) or the study did not report independent evaluation of COX-2 staining by two assessors (*n*=1). Five studies met the criteria for inclusion and data were extracted to conduct a meta-analysis. Two studies were undertaken in the United States of America, one in Italy, one in Spain and one in Korea (Table 1). One study was population based (Kerlikowske *et al*, 2010) and the remaining studies used samples obtained from hospital archives. The earliest samples included were obtained between 1983 and 1994 in Kerlikowske *et al*'s study and the most recent samples included were obtained between 1998 and 2003 in a study by Perrone *et al* (2005). The random effects pooled estimate of COX-2 positivity was 53% (95% CI, 44–61; *P*=0.04) with evidence of moderate heterogeneity *I*^2^=60.1% (Figure 1).

### DCIS adjacent to invasive cancer

Five of the 36 studies that examined DCIS investigated DCIS adjacent to invasive cancer. Four of these were excluded as the study did not use monoclonal anti-COX-2 antibodies (*n*=1) or did not report independent evaluation by two assessors (*n*=3). Therefore only one hospital-based study (undertaken in Germany) met the inclusion criteria. The time period over which samples were obtained was not specified. The frequency of COX-2 expression in this study was 55.2% (Table 1).

### MICB

The search identified 18 studies (Medline *n*=1, Embase *n*=1 and Web of Science *n*=16). After removal of duplicates, 16 articles were screened. Additionally, one paper was included from the DCIS search results. Of these, four potentially relevant studies were fully screened. Three studies were excluded as COX-2 expression was not examined (*n*=2) or the study did not state use of monoclonal anti-COX-2 antibodies (*n*=1). One study (undertaken in Spain) met the inclusion criteria. This was a hospital-based study and samples were extracted from tissue archives. The MICB samples were retrieved between 1985 and 2003. The frequency of COX-2 expression was 74.0% (Table 1).

### Invasive breast cancer

**Figure 2:**
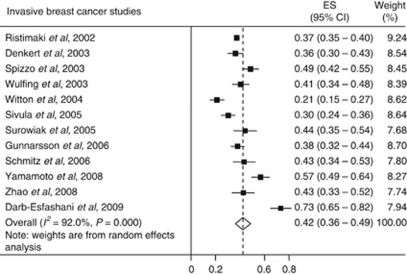
Forest plots of COX-2 expression in invasive breast cancer.

The search identified 5204 studies (Medline *n*=728, Embase *n*=1142 and Web of Science *n*=3334). After removal of duplicates, 4914 articles were screened. Of these, 154 potentially relevant papers were fully screened. In all, 142 studies were excluded as the proportion (%) of COX-2 expression in invasive breast cancer was not reported (*n*=50), the sample size was below 100 (*n*=33), the study used cell lines or animal models (*n*=22), the study included previous/adjoining cancers or other tissue samples (*n*=13), the study did not report independent evaluation by two assessors (*n*=7), the paper was a review (*n*=6), male patients were included in the study (*n*=5), no translation was available for the paper (*n*=2), the study did not use monoclonal anti-COX-2 antibodies (*n*=2), the tissue source was used in a larger study (*n*=1) or the paper had missing data (*n*=1). A total of 12 studies met the criteria for inclusion and data were extracted to conduct a meta-analysis. Four studies were undertaken in Germany, two in Finland, one from each of Austria, Japan, Poland, Sweden, the United Kingdom and the United States of America (Table 1). One study was population based (Ristimaki *et al*, 2002) and the remaining studies used samples obtained from hospital archives. The earliest samples were obtained between 1977 and 1990 in the study by Gunnarsson *et al* (2006) and the most recent samples were obtained between 1993 and 2001 in the study by Yamamoto *et al* (2008). The individual study estimates were widely dispersed about the random effects pooled estimate; 42% (95% CI, 36–49; *P*<0.001) with evidence of high heterogeneity *I*^2^=92.0% (Figure 2).

## Discussion

The aim of this systematic review was to determine the frequency of COX-2 expression in the continuum from normal breast epithelium to invasive carcinoma of the breast. A robust estimate could not be obtained for normal breast epithelium, DCIS adjacent to invasive breast cancer or MICB as so few studies have been performed. COX-2 expression was common in DCIS and invasive breast cancer (pooled estimates in the studies of 53% and 42% respectively), and there was no significant difference in prevalence of COX-2 expression between these lesions.

To our knowledge this is the largest, and first systematic, review of publications reporting COX-2 expression in invasive breast cancer. A review by Denkert *et al* (2004), which did not include a meta-analysis, investigated COX-2 expression in 2392 primary breast cancers by IHC and reported COX-2 positivity of 40%. We included 12 studies that reported on COX-2 expression in 3492 invasive breast cancer samples. The overall COX-2 expression from our meta-analysis in invasive breast cancer was consistent with findings of the Denkert review. In addition, our review has been the first to review COX-2 expression in DCIS samples separate to invasive breast cancer. We can report the prevalence of COX-2 expression in DCIS to be similar to that of invasive breast cancer. It is believed that most invasive breast carcinoma originates from DCIS and that the two coexist in ∼50% of cases (Boland *et al*, 2004). Only one study (Leo *et al*, 2006) met our inclusion criteria in DCIS-adjoining invasive breast cancer. The frequency of COX-2 expression reported in this study (study estimate 55.2%) was similar to that of DCIS or invasive breast cancer in our meta-analyses. One study (de la Torre *et al*, 2010) met the inclusion criteria in MICB. This study reported COX-2 expression in 74% of samples. However, the paucity of studies in this tissue type has prevented any COX-2 frequency to be established. The two studies of normal breast epithelium included in our review provided very different estimates of the prevalence of COX-2 expression in normal tissue, so we cannot determine whether COX-2 expression occurs more or less frequently in invasive or *in-situ* breast cancer than in normal breast tissue.

We adopted a number of approaches to increase the robustness of the results of this review/meta-analysis. Studies with fewer than 10 normal breast epithelium, DCIS or MICB samples and 100 invasive breast cancer samples were excluded, only samples which expressed COX-2 at a moderate or higher level (reported by two observers) were considered positive and only studies that used monoclonal anti-COX-2 antibodies were included in an attempt to limit between-study variation. However, high heterogeneity was still evident despite our strict exclusion criteria. Between-study variance may have resulted from the diverse populations included in the studies. In addition, differences in the evaluation of COX-2 expression and cut-off marks for COX-2 positivity may have caused variation in individual study estimates. The evaluation of COX-2 expression by quantity of staining alone may allow more tissue to be regarded as positively stained, as this does not incorporate the intensity of staining like in other evaluation methods. Finally, several of the studies included were small and only investigated archived tissue from single centres. Larger population-based studies would provide more robust findings.

Several studies have reported on COX-2 expression and disease recurrence in invasive breast cancer and have shown elevated levels of COX-2 expression to be associated with a more aggressive cancer and decreased survival (Ristimaki *et al*, 2002; Haffty *et al*, 2008). Further, three observational studies have shown a reduced risk of disease progression in breast cancer survivors who use NSAIDs (Blair *et al*, 2007; Kwan *et al*, 2007; Holmes *et al*, 2010). However, few studies have provided follow-up information on the relationship between COX-2 positivity in DCIS and disease progression after treatment, and none have explored whether NSAID use modulates the risk of progression (Kulkarni *et al*, 2008; Kerlikowske *et al*, 2010).

This review has shown high (and similar) levels of COX-2 expression in DCIS and invasive breast cancer. This suggests that COX-2 is involved in early breast cancer carcinogenesis. Further investigation of the importance of COX-2 expression and inhibition in the progression of DCIS is warranted.

## Figures and Tables

**Table 1:** Characteristics of the included studies for normal breast epithelium, DCIS, DCIS-adjoining invasive breast cancer, MICB and invasive breast cancer

**Study**	**Location**	**Number of samples included (n)**	**COX-2 positive (n)**	**COX-2 positive (%)**
*Normal breast*				
Cho *et al* (2006)	Korea	15	1	6.7
Zhao *et al* (2008)	USA	19	19	100.0
				
*DCIS*				
Perrone *et al* (2005)	Italy	49	31	63.3
Cho *et al* (2006)	Korea	30	19	63.3
Kulkarni *et al* (2008)	USA	59	27	45.8
de la Torre *et al* (2010)	Spain	52	28	53.8
Kerlikowske *et al* (2010)	USA	279	124	44.4
				
*DCIS-adjoining invasive breast cancer*				
Leo *et al* (2006)	Germany	29	16	55.2
				
*MICB*				
de la Torre *et al* (2010)	Spain	40	30	74.0
				
*Invasive breast cancer*				
Ristimaki *et al* (2002)	Finland	1576	589	37.4
Denkert *et al* (2003)	Germany	221	80	36.2
Spizzo *et al* (2003)	Austria	212	103	48.6
Wulfing *et al* (2003)	Germany	192	78	40.6
Witton *et al* (2004)	UK	179	38	21.2
Sivula *et al* (2005)	Finland	231	70	30.3
Surowiak *et al* (2005)	Poland	104	46	44.2
Gunnarsson *et al* (2006)	Sweden	284	108	38.0
Schmitz *et al* (2006)	Germany	113	49	43.4
Yamamoto *et al* (2008)	Japan	171	97	56.7
Zhao *et al* (2008)	USA	108	46	42.6
Darb-Esfahani *et al* (2009)	Germany	101	74	73.3

Abbreviations: COX2=cyclooxygenase 2; DCIS=ductal carcinoma *in situ* of breast; MCIB=microinvasive carcinoma of the breast.

## Supplementary Material

Supplementary Online Resource 1

Supplementary Online Resource 2
