# Supplementary material for: A systematic review to establish the frequency of cyclooxygenase-2 expression in normal breast epithelium, ductal carcinoma in situ, microinvasive carcinoma of the breast and invasive breast cancer
Source: Br J Cancer. 2011 Jun 7;105(1):13–7. doi: 10.1038/bjc.2011.204 (PMC3137418; doi:10.1038/bjc.2011.204)
Supplement: Supplementary Online Resource 1 [file bjc2011204x1.doc]

**A systematic review to establish the frequency of cyclooxygenase-2 expression in normal breast epithelium, ductal carcinoma in situ, microinvasive carcinoma of the breast and invasive breast cancer**

Janine A. Glover • Carmel M. Hughes • Marie M. Cantwell^1^ • Liam J. Murray

Centre for Health Improvement, Queen’s University, Belfast, Northern Ireland, United Kingdom

^1^Centre for Public Health, Queen’s University, Belfast, Northern Ireland, United Kingdom

Janine A. Glover *corresponding author

E-mail: jglover02@qub.ac.uk

**Online Resource 1**

This search strategy was produced in order to identify primary studies containing at least one key word or Medical Subject Heading (MeSH) term from each of the following expressions:

1. cyclo(-)oxygenase-2 OR COX-2 OR cyclo(-)oxygenase ii OR COX-ii

AND

Normal breast epithelium

1. normal breast tissue OR normal breast epithelium OR breast tissue OR breast epithelium OR breast reduction OR mammoplasty

Or

DCIS

1. ductal carcinoma in situ OR Carcinoma, Intraductal, Noninfiltrating OR DCIS OR intraductal carcinoma in situ OR pre(-)invasive ductal carcinoma(s) OR non(-)invasive ductal carcinoma(s) OR pre(-)invasive breast cancer(s) OR non(-)invasive breast cancer(s) OR stage zero breast cancer(s) OR non(-)infiltrating intraductal carcinoma(s) OR intra(-)ductal carcinoma(s) OR lobular carcinoma in situ OR LCIS OR lobular intra(-)epithelial neoplasia(s) OR LIN OR mammary intra(-)epithelial neoplasia(s) OR ductal intra(-)epithelial neoplasia(s) OR ductal neoplasm(s) OR ductal neoplasia(s) OR DIN OR non(-)invasive breast tumo(u)r(s) OR pre(-)invasive breast tumo(u)r(s) OR non(-)infiltrating carcinoma(s)

Or

MICB

1. Microinvasive carcinoma of the breast OR MICB OR ductal carcinoma in situ with microinvasion OR DCIS-MI OR microinvasive carcinoma

Or

Invasive breast cancer

1. Invasive breast cancer OR invasive breast carcinoma OR breast cancer OR breast neoplasm(s) OR breast tumo(u)r(s) OR cancer of the breast OR ductal carcinoma(s) OR lobular carcinoma(s) OR malignant neoplasm(s) of the breast OR malignant tumo(u)r(s) of the breast OR mammary cancer(s) OR mammary carcinoma(s)
