# Supplementary material for: A systematic review to establish the frequency of cyclooxygenase-2 expression in normal breast epithelium, ductal carcinoma in situ, microinvasive carcinoma of the breast and invasive breast cancer
Source: Br J Cancer. 2011 Jun 7;105(1):13–7. doi: 10.1038/bjc.2011.204 (PMC3137418; doi:10.1038/bjc.2011.204)
Supplement: Supplementary Online Resource 2 [file bjc2011204x2.doc]

**A systematic review to establish the frequency of cyclooxygenase-2 expression in normal breast epithelium, ductal carcinoma in situ, microinvasive carcinoma of the breast and invasive breast cancer**

Janine A. Glover • Carmel M. Hughes • Marie M. Cantwell^1^ • Liam J. Murray

Centre for Health Improvement, Queen’s University, Belfast, Northern Ireland, United Kingdom

^1^Centre for Public Health, Queen’s University, Belfast, Northern Ireland, United Kingdom

Janine A. Glover *corresponding author

E-mail: jglover02@qub.ac.uk

**Online Resource 2**

Varying methods for COX-2 expression evaluation were used in the studies included in our analyses. In order to report COX-2 expression across these studies in a consistent manner, only lesions reported to express COX-2 at a moderate or strong level were defined as COX-2 positive. We assessed individual evaluation methods to determine the COX-2 positivity as follows.

An immunoreactive score (IRS) defines COX-2 expression in a lesion by multiplying a score for staining intensity (0-3 or 4) with a score for quantity of staining (0-3 or 4). Included studies adopted the following methods. An IRS score of 9-12/12 as strong, 5-8/12 as moderate and 0-4/12 as weak; Cho *et al*, 2006; Leo *et al*, 2006; Perrone *et al*, 2005 and Spizzo *et al*, 2003. Within our review scores of ≥5 were considered as moderate to strong. An IRS score of 7-12/12 as COX-2 positive and 0-6/12 as negative was also used; Darb-Esfashani *et al*, 2009 and Denkert *et al*, 2003. Within our review a positive score of 7-12 was considered to have a moderate to strong COX-2 expression.

Another method used reported absent staining as 0; weak as 1; moderate as 2 and strong as 3 based on the extent and intensity of staining with moderate and strong regarded as positive; De la Torre *et al*, 2010. Within our review a score of ≥2 was considered to be moderate/strong and equated to positive COX-2 expression.

One study used the Allred class with staining (extent and intensity) categorised from 0-4 and score of 2-4 regarded as over-expressing; Kerlikowske *et al*, 2010. Within our review a score ≥2 was considered to be moderate to strong COX-2 expression.

Other evaluation methods assessed only quantity of COX-2 staining.

Absent of COX-2 staining as 0; weak expression showing less than 10% staining as 1+; moderate to strong expression showing 10-90% of cells as 2 and strong with greater than 90% cells intensely stained as 3; Ristimaki *et al*, 2002; Sivula *et al*, 2005 and Wulfing *et al*, 2003. Within our review a score ≥2 was considered to be moderate to strong COX-2 expression.

COX-2 positivity score of ≥ 1 or 10% positivity, whereby any samples with the quantity of staining greater than 1 or 10% of total cells showing staining were defined as being COX-2 positive; Kulkarni *et al*, 2008; Schmitz *et al*, 2006; Surowiak *et al*, 2005 and Yamamoto *et al*, 2008. Within our review a score regarded as positive was considered to be moderate to strong COX-2 expression.

A score based on (0 x percentage of cells not stained) + (1 x percentage of cells weakly stained) + (3 x percentage of cells strongly stained). A score in the sample tissue with a greater expression than the median COX-2 expression in adjacent normal breast tissue was described as over-expressing; Witton *et al*, 2004. Within our review a score above the median COX-2 expression of the adjacent normal breast tissue was considered to have a positive COX-2 expression.

Finally, two papers categorised lesions only as no staining, weak, moderate and positive; Gunnarsson *et al*, 2006 and Zhao *et al*, 2008. We included moderate and positive categories within our review.
